# The risks of long-term re-injection in supercritical geothermal systems

**DOI:** 10.1038/s41467-019-12146-0

**Published:** 2019-09-26

**Authors:** Francesco Parisio, Victor Vilarrasa, Wenqing Wang, Olaf Kolditz, Thomas Nagel

**Affiliations:** 1 Chair of Soil Mechanics and Foundation Engineering, Technische Universitaet Bergakademie Freiberg, Gustav-Zeuner-Strasse 1, Zimmer 108, 09599 Freiberg, Germany; 2 Department of Environmental Informatics, Helmholtz Centre for Environmental Research–UFZ, Permoser Str. 15, 04318 Leipzig, Germany; 3 Institute of Environmental Assessment and Water Research (IDAEA), Spanish National Research Council (CSIC), Jordi Girona 18-26, 08034 Barcelona, Spain; 4 Associated Unit: Hydrogeology Group UPC-CSIC, Jordi Girona 1-3, 08034 Barcelona, Spain; 5 Applied Environmental Systems Analysis, Technische Universitaet Dresden, 01062 Dresden, Germany

**Keywords:** Geophysics, Seismology

## Abstract

Supercritical geothermal systems are appealing sources of sustainable and carbon-free energy located in volcanic areas. Recent successes in drilling and exploration have opened new possibilities and spiked interest in this technology. Experimental and numerical studies have also confirmed the feasibility of creating fluid conducting fractures in sedimentary and crystalline rocks at high temperature, paving the road towards Enhanced Supercritical Geothermal Systems. Despite their attractiveness, several important questions regarding safe exploitation remain open. We dedicate this manuscript to the first thermo-hydro-mechanical numerical study of a doublet geothermal system in supercritical conditions. Here we show that thermally-induced stress and strain effects dominate the geomechanical response of supercritical systems compared to pore pressure-related instabilities, and greatly enhance seismicity during cold water re-injection. This finding has important consequences in the design of Supercritical Geothermal Systems.

## Abstract (web summary)

Can we pump water into deep active volcanic areas? Here, the authors model the effect of water circulation into supercritical geothermal systems and show that the effect of rock cooling dominates the seismicity increase over the pore pressure changes.

## Introduction

Enhanced Supercritical Geothermal Systems (ESGS) are still widely unexplored and fundamental questions are not only to be answered, but to be formulated in the first place. Provided it is possible to stimulate permeability of the reservoirs, is it feasible to circulate fluids between two wells as in traditional Geothermal Systems (GS)? A positive answer to this question entails the second one: can fluid circulation be maintained safely and what is the potential for fluid injection-induced seismicity in ESGS? Additionally, what could be the effect of reservoir cooling by cold water re-injection? Addressing these questions is crucial for the successful development of ESGS.

The last two decades have witnessed an increasing research and industrial interest towards geothermal energy projects in magmatic environments^1–8^: as a consequence of high temperature and depth, fluids can be found in supercritical (SC) conditions, which for pure water occurs at temperature $T{\,}> {\,}37{4}{\,}^{\circ }{\rm{C}}$ and pressure $p{\,}> {\,}22.064$ MPa (http://www.iapws.org/). The idea of producing geothermal power by drilling directly into magmatic environments dates back more than a decade with pioneering projects like the Icelandic Deep Drilling Projects (IDDP1 and IDDP2), at the Krafla^1^ and Reykjanes^9^ fields, in Iceland. Producing SC geothermal fluids can have enormous advantages: the high enthalpy per unit mass implies up to a tenfold increase in power generation^2^ and low fluid viscosity implies greatly enhanced hydraulic conductivity. The latter favours advective heat transport and diminishes pressure build-up. Only few wells worldwide have encountered SC fluid conditions^6,7^ and current research projects are located in active volcanic areas such as Kakkonda, Japan^10^, Taupo Volcanic Area, New Zealand^11^, Larderello, Italy^12,13^, Krafla and Reykjanes, Iceland^14,15^, The Geysers and Salton Sea, the USA^16–18^ and Los Humeros, Mexico^19–22^.

**Fig. 1:**
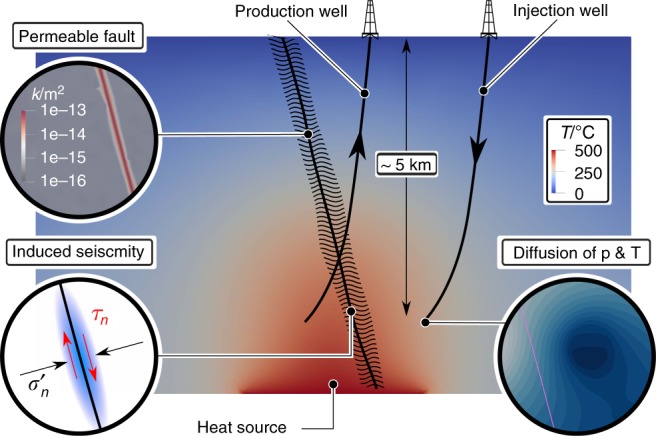
Enhanced Supercritical Geothermal System. Schematic representation of a doublet system in an Enhanced Supercritical Geothermal System (ESGS), with pre-existing magmatic heat source that generates a convective hydrothermal circulation. A doublet system of injection/production alters the pressure and temperature fields, leading to potential fault instability

Volcanic formations at depth are usually data-poor environments as a consequence of the technical difficulties associated with drilling exploration wells in high-temperature and highly corrosive environments. Indirect geophysical methods^5,21^ in combination with advanced numerical simulations^4,8^ can overcome the lack of information and provide preliminary assessments of the deep thermal structures and convective anomalies. Alongside, laboratory experiments are essential to build realistic geomechanical models based on insights from the rheological behaviour of rocks at high pressure and temperature and its effect on fluid circulation^23–26^. Close to the brittle-ductile transition conditions of pressure and temperature, new findings suggest that fractures are sufficiently permeable to allow fluid circulation^27^ and, in case of insufficient fracture density, enhancement strategies are likely to be successful^28^, proving that ESGS development in low-permeable reservoirs close to the ductile crust is possible (Fig. 1). Some insights can be gained from Enhanced Geothermal Systems (EGS), in which fluid is injected in two stages^29^: a first stage in which permeability enhancement is achieved by hydro-fracturing and/or hydro-shearing, chemical or thermal stimulation, and a second stage where fluids are continuously produced and re-injected to facilitate advective heat transport and reach fluid mass balance. The injection of fluids^30–35^ and the associated effects of reservoir cooling^30,34,36^ are linked with an increased likelihood of inducing seismic events, an occurrence that in the past has jeopardised several geothermal projects, sometimes leading to the suspension of all operations^37–43^. During stimulation injection, micro-seismicity is associated with hydro-fracture propagation and larger events happen during prolonged injection^44^. Nevertheless, there are reported cases in which $M{\,}> {\,}5$ earthquakes were likely triggered during the stimulation phase of deep geothermal systems^40,42,43^. In this study, we focus exclusively on the prolonged fluid re-injection and assume a successful stimulation of the wells’ surroundings already took place. Although the connection between cooling and reservoir stability is known and numerical studies applied to traditional geothermal systems have highlighted the influence of thermal gradients^45^, fault and rock permeability^46^, stress regime^30^, competition between thermal and hydraulic processes^47^ and tensile fracturing effects on permeability^48^, the issue has never been addressed in ESGS systems. Specifically, cooling in ESGS could take on a whole new dimension, as the potential temperature drop and associated thermal stress variation can be very high. The complexity and strong non-linearity of the processes involved in ESGS make them unique environments which require state-of-the-art numerical analyses to understand the main physical mechanisms controlling their geomechanical behaviour.

Here, we investigate for the first time the feasibility and safety of prolonged fluid production and re-injection in ESGS by performing coupled thermo-hydro-mechanical non-linear analyses with innovative features such as poro-mechanical changes, porosity-dependent permeability, full range of water equation of state, non-linear fluid compressibility, fault stability and rate of seismic production. We have developed a full model of the reservoir ranging from surface to 7 km depth, applied an extreme cooling scenario considering changes in the water properties during cold water injection and provided interpretations in terms of seismicity increase and fault reactivation. We have found that seismicity increases mainly as a consequence of the thermal quenching effects and that pore pressure increments play a secondary role in controlling rock and fault stability. The thermal effects are delayed in time because of the slower heat advection processes compared to pressure diffusion. The increased viscosity during cold fluid re-injection plays a secondary role compared to thermal quenching. This work constitutes the first of a kind numerical study of the complex geomechanical processes induced in ESGS.

## Modelling approach

### Theoretical model

**Fig. 2:**
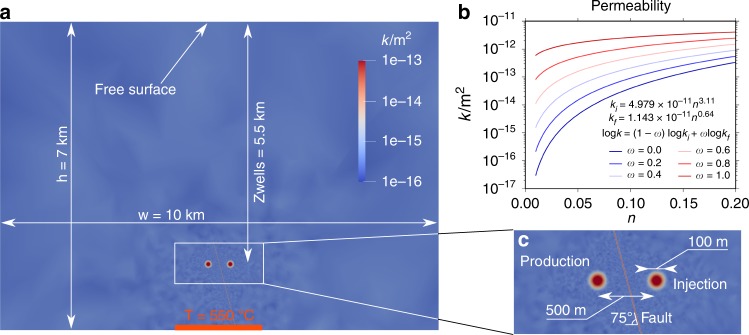
Numerical model and equivalent permeability. **a** Model setup of the complete reservoir for initialisation phase, in which a constant bottom temperature is applied to initialise the thermal plume. **b** Permeability curves of volcanic rocks are function of porosity^50^ in a logarithmic relation with $\omega$ representing the degree of fracturing of the rock. **c** Detail of the geothermal doublet located at 5.5 km depth with the fault between the wells and the distribution of initial permeability for the injection phase

We have employed transient numerical finite element analyses of coupled thermo-hydro-mechanical (THM) processes to investigate an idealised ESGS systems formed by an injection and a production well (Fig. 2a). The non-linear system of partial differential equations describing the mass, energy and momentum balance of porous media is^49^

1 $$\begin{array}{*{20}{l}} \left(n{\beta }_{\text{w}}+\frac{\alpha -n}{{K}_{\text{s}}}\right)\frac{{\rm{d}}_{\rm{s}}p}{{\rm{d}}t}-\left[n{\alpha }_{\text{w}}+3{\alpha }_{\text{s}}\left(n-1\right)\right]\frac{{\rm{d}}_{\rm{s}}T}{{\rm{d}}t}\hfill\\ \quad- \, \nabla \cdot \left[\frac{{\bf{k}}}{{\mu }_{{\rm{w}}}}\left(\nabla p-{\rho }_{{\rm{w}}}{\bf{g}}\right)\right]+\alpha \nabla \cdot \frac{{\rm{d}}_{\rm{s}}{\bf{u}}}{{\rm{d}}t}={Q}_{{\rm{H}}}\hfill\\ \hskip -10pt \quad\left[n{\rho }_{{\rm{w}}}{c}_{{\rm{w}}}+(1-n){\rho }_{{\rm{s}}}\ {c}_{{\rm{s}}}\right]\frac{{\rm{d}}_{\rm{s}}T}{{\rm{d}}t}-\nabla \cdot \left[\left(n{\lambda }_{{\rm{w}}}{\bf{I}}+(1-n){{\boldsymbol{\lambda }}}_{{\rm{s}}}\right)\nabla T\right]\hfill\\ \quad- \,\, {\rho }_{{\rm{w}}}{c}_{{\rm{w}}}\frac{{\bf{k}}}{{\mu }_{{\rm{w}}}}\left(\nabla p-{\rho }_{{\rm{w}}}{\bf{g}}\right)\cdot \nabla T={Q}_{{\rm{T}}}\hfill\\ \hskip -8pt\quad\frac{E}{2\left(1-2\nu \right)\left(1+\nu \right)}\nabla \left(\nabla \cdot {\bf{u}}-3{\alpha }_{{\rm{s}}}\Delta T\right)+\frac{E}{\left(1-2\nu \right)}{\nabla }^{2}{\bf{u}}-\nabla \cdot \left(\alpha p{\bf{I}}\right)\hfill\\ \quad+\left[n{\rho }_{\text{w}}+(1-n){\rho }_{\text{s}}\right]{\bf{g}}={\bf{0}},\hfill\end{array}$$

and is formulated in terms of primary variables describing the fields of pore pressure $p$, temperature $T$ and displacement ${\bf{u}}$. $t$ is time and ${\bf{k}}$ is the permeability tensor. Subscripts ‘s’ and ‘w’ stand for solid and for water, respectively, and ${\bf{I}}$ is the identity tensor. The fractured rock properties are set to values typical of a crystalline rock with porosity $n=0.01$, solid density ${\rho }_{{\rm{s}}}=2700\ {\rm{kg}}\ {{\rm{m}}}^{-3}$, linear thermal expansion coefficient ${\alpha }_{\text{s}}=1\cdot 1{0}^{-5}\ {\,}{{\rm{K}}}^{-1}$, specific heat capacity ${c}_{{\rm{s}}}=950\ {\rm{J}}{\,} {{\rm{kg}}}^{-1}{\,}{{\rm{K}}}^{-1}$, thermal conductivity ${{\boldsymbol{\lambda }}}_{{\rm{s}}}=3{\bf{I}}\ {\rm{W}}{\,} {{\rm{m}}}^{-1}{\,}{{\rm{K}}}^{-1}$, Young’s modulus $E=60$ GPa, Poisson’s ratio $\nu {\,}={\,}0.25$ and Biot’s coefficient $\alpha =1{\,}-{\,}K/{K}_{\text{s}}{\,}={\,}0.5$, where K is the drained bulk modulus and ${K}_{\text{s}}$ is the intrinsic bulk modulus of the solid phase. The gravity acceleration vector is indicated by ${\bf{g}}$ and the source terms for the thermal and hydraulic problem are ${Q}_{{\rm{T}}}$ and ${Q}_{{\rm{H}}}$, respectively. The fault has different mechanical properties with $n{\,}={\,}0.05$, $E{\,}={\,}20$ GPa and $\alpha {\,}={\,}1.0$. The equation of state (EOS) of water is taken after the IAPWS-IF97 standards using the freesteam application (http://freesteam.sourceforge.net/) and properties of water like density ${\rho }_{{\rm{w}}}$, dynamic viscosity ${\mu }_{{\rm{w}}}$, specific heat capacity ${c}_{{\rm{w}}}$, thermal conductivity ${\lambda }_{{\rm{w}}}$, compressibility ${\beta }_{\text{w}}$ and thermal expansion ${\alpha }_{\text{w}}$ are functions of its thermodynamic state (see Methods for the computation of fluid compressibility and thermal expansion).

The relation between permeability evolution and porosity is still a matter of deep research and debate in literature. Employing the formulation of Kozeny–Carman^51,52^, in which permeability is proportional to the cube of porosity $k{\,}\propto{\,} {n}^{3}$, is common practice^53^. The Kozeny–Carman relationship, which is formulated for flow in given shape channels^54^ or assemblies of perfect spheres, takes into account tortuosity in the flow but becomes inaccurate for fractured media. Recent research empirically linked permeability of fully fractured ${k}_{\text{f}}$ and intact ${k}_{\text{i}}$ rock to porosity $n$, based on permeability measurements on 70 different samples of volcanic and magmatic rocks^50^. We have introduced a formulation that interpolates between intact ${k}_{\text{i}}$ and fractured ${k}_{\text{f}}$ rock permeability as $\mathrm{log}{\,}k{\,}={\,}\left(1-\omega \right){\,}\mathrm{log}{\,}{k}_{\text{i}}+\omega{\,} \mathrm{log}{\,}{k}_{\text{f}}$, in which $\omega$ is a newly defined parameter representing either fully fractured ($\omega {\,}={\,}1$) or intact ($\omega {\,}={\,}0$) rock (Fig. 2b).

The Coulomb Failure Stress ${\rm{CFS}}{\,}={\,}|{\tau }_{{\rm{n}}}{\,}|+\mu {\sigma }^{\prime }_{{\rm{n}}}$ is suited to estimate potential failure^34^, where ${\tau }_{{\rm{n}}}$ and ${\sigma }_{{\rm{n}}}^{{\prime} }$ are, respectively, the shear and the effective normal stress acting on a given plane. We consider the orientation of the main fault (i.e., dipping $7{5}^{\circ }$) and a frictional coefficient $\mu {\,}={\,}0.577$, which corresponds to a friction angle $\phi {\,}={\,}3{0}^{\circ }$. $\Delta {\rm{CFS}}{\,}> {\,}0$ indicates increased instability in fractures and faults with that given orientation. The mobilised friction coefficient, which indicates the proximity of the stress state to failure conditions, is ${\mu }_{{\rm{fr}}}{\,}={\,}{\tau }_{{\rm{n}}}/\left(-{\sigma }^{{\prime}}_{{\rm{n}}}\right)$. The Drucker–Prager criterion is an alternative to estimate the failure potential in terms of invariants of the stress tensor (direction independence)

2 $${q}_{\text{dp}}=\frac{6\sin{\,} \phi }{3-\sin{\,} \phi }\left({-\sigma}_m ^{\prime} \right)+\frac{6c^{\prime} \cos{\,} \phi }{3-\sin{\,} \phi }$$

where $c^{\prime} {\,}={\,}30$ MPa is the cohesion of the fractured rock, ${q}_{\text{dp}}$ is the Drucker–Prager deviatoric stress, $\sigma ^{\prime}_{\rm{m}} {\,}={\,}{\rm{tr}}{\,} \left({\boldsymbol{\sigma }}^{\prime} \right)/3$ is the mean effective stress, ${\boldsymbol{\sigma }}^{\prime} {\,}={\,}{\boldsymbol{\sigma }}{\,}+{\,}\alpha p{\bf{I}}$ is the effective stress tensor. The mobilised failure ratio for the Drucker–Prager model is ${M}_{\text{DP}}{\,}={\,}{q}_{\text{dp}}/q$, where $q{\,}={\,}\sqrt{3{\,}\left({\bf{s}}:{\bf{s}}\right)/2}$ is the acting deviatoric stress and ${\bf{s}}{\,}={\,}{\boldsymbol{\sigma }}^{\prime}{\,} -{\,}{\bf{I}}{\sigma}_m ^{\prime}$.

The prediction model of time-dependent earthquake nucleation in injection-induced seismicity is based on rate and state theory of friction on faults^55,56^. The equation for the rate of seismic production relative to the background seismic noise, $R$, writes $\dot{R}{\,}={\,}R/{t}_{{\rm{a}}}\left({\dot{\tau }}_{{\rm{c}}}/{\dot{\tau }}_{0}{\,}-{\,}R\right)$, where ${t}_{{\rm{a}}}{\,}={\,}A{\sigma }_{{\rm{n}}}^{{\prime} }/{\dot{\tau }}_{0}$ is a characteristic time, $A$ is a constitutive parameter and equals $0.02$ for hydrothermal systems^55^, ${\dot{\tau }}_{0}{\,}={\,}1{\,}\times{\,} 1{0}^{-3}{\,} {\rm{MPa}}{\,} {{\rm{yr}}}^{-1}$ is the background shear stressing rate, ${\tau }_{{\rm{c}}}{\,}={\,}\text{CFS}$ and the time derivative symbol is $\dot{\text{x}}{\,}={\,}{\rm{d}}\text{x}/{\rm{d}}t$.

### Numerical model setup

The numerical solution is obtained via the finite element method (FEM) with a Lagrangian approach with respect to the solid. The model is implemented in the C++, object-oriented and open-source finite element solver OpenGeoSys^57^. We have built a representative FEM model of a typical deep SC geothermal system with a two-dimensional plane strain numerical model 7 km deep and 10 km wide (Fig. 2a). We first simulate an initialisation phase to reach the conditions of a SC reservoir located above a magmatic intrusion. The bottom central part of the model is assumed to be at a constant temperature of $55{0}{\,}^{\circ }{\rm{C}}$ over a width of 2 km. The hydraulic conditions are no-flow at the bottom, hydrostatic pressure at the sides and a fixed atmospheric pressure at the top. The thermal conditions at the sides are fixed temperature with the average gradient of $3{0}{\,}^{\circ }{\rm{C}}{\,} {{\rm{km}}}^{-1}$. The initialisation phase is performed in hydro-thermal conditions. The model is solved until temperature and pore pressure profiles are reasonably close to what are believed to be representative conditions of a SC reservoir^8,17,58–61^.

The injection phase, in which the coupled THM system of Eq. (1) is solved, uses the $p$ and $T$ distribution obtained at the end of the initialisation phase. The initial stress state is given by a lithostatic vertical stress ${\sigma }_{V}$ corresponding to a solid density of $2700\ {\,}{\rm{kg}}{\,} {{\rm{m}}}^{-3}$ and a normal faulting stress regime, i.e., ${\sigma }_{V}{\,}> {\,}{\sigma }_{H}{\,}={\,}{\sigma }_{h}$, with ${\sigma }_{H}/{\sigma }_{V}{\,}={\,}0.65$. The wells, which are spaced 500 m horizontally, are located at 5.5 km depth and are represented as points (Fig. 2c). A permeable (${\bf{k}}{\,}={\,}1.0{\,}\times{\,} 1{0}^{-13}{\bf{I}}{\,} {{\rm{m}}}^{-2}$) inclined fault with a dip of $7{5}^{\circ }$ and a thickness of 1 m is placed between the two wells. Rock permeability depends on the fracture density $\omega$ (Fig. 2b), which follows the distribution indicated in Fig. 2c. We hypothesise a radius of 100 m in which, through stimulation (not modelled here), a permeability increase of up to three orders of magnitude around the two wells was reached prior to injection.

To estimate the downhole injection temperature difference $\Delta T$, because of data scarcity in SC reservoirs, we have extrapolated values from records on existing wells at lower temperature^62^, leading to $\Delta T{\,}={\,}32{2}{\,}^{\circ }{\rm{C}}$ for an initial reservoir temperature of $45{7}{\,}^{\circ }{\rm{C}}$ (see Table 1). Thus, we have run 2 different cases of injection temperature ($\Delta T{\,}={\,}30{0}{\,}^{\circ }{\rm{C}}$ and $\Delta T{\,}={\,}0$ °C) with constant injection and production volumetric rate ${q}_{\text{v}}{\,}={\,}8.91\times 1{0}^{-5}{\,} {{\rm{m}}}^{3}{\,} {{\rm{s}}}^{-1}$, in the hypothesis of an injection distributed over a 300 m section of open well, and for a total of 25 years of exploitation.

**Table 1:** Estimate of the injection temperature, obtained by extrapolation of values compiled in a literature review of several geothermal wells^62^. The linear extrapolation from literature values (with the estimated average temperature of Low Enthalpy systems of $14{0}\,^{\circ}{\rm{C}}$) leads to the value of $\Delta T\,=\,32{2}\,^{\circ }{\rm{C}}$ for the SC case

Category	$${T}_{\text{min}}$$	$${T}_{\text{max}}$$	$$< {T} > $$	$$\Delta T$$
	${}^{\circ }{\rm{C}}$	${}^{\circ }{\rm{C}}$	${}^{\circ }{\rm{C}}$	${}^{\circ }{\rm{C}}$
Hot water	$-$	$220$	$140$	$55$
Low enthalpy	$220$	$250$	$235$	$131$
Medium enthalpy	$250$	$300$	$275$	$186$
High enthalpy	$250$	$330$	$290$	$169$
Supercritical	$-$	$-$	$457$	$322$

## Results

### Initial conditions of the reservoir

**Fig. 3:**
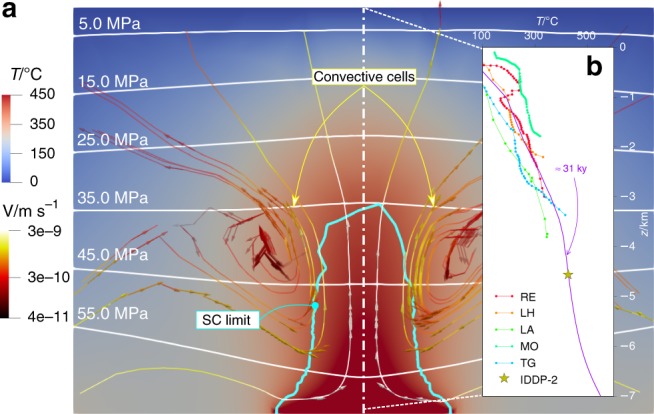
Initial conditions in the reservoir. **a** The solution of the hydro-thermal (TH) problem refers to a time of roughly 31’000 yr. Instability triggers two convective cells and supercritical resources are found in the deeper area and are delimited with a cyan line. **b** The temperature profile is compared with several geothermal wells in the world close to or at supercritical conditions, such as Reykjanes (RE)^59^, Los Humeros (LH)^58^, Larderello (LA)^61^, Mofete (MO)^60^, The Geysers (TG)^17^ and IDDP-2 at Reykjanes^9^

The simulated SC geothermal reservoir presents a density-driven instability in the form of two convective cells with upward flow at the centre of the model, where the heat source is located (Fig. 3a). SC water is located close to the deep magmatic source at a depth between 4 and 7 km. The maximum flow velocity, which is located within the SC region and drives the thermal plume upward, enhances the advective component of heat transport and is responsible for the pressure drop above the heat source, resulting in a sub-hydrostatic pore pressure at the centre of the model. The results are in agreement with previous computations of deep SC geothermal systems^8^. The temperature distribution along depth at the centre of the model compares well with the one of worldwide SC geothermal sites^9,17,58–61^ (Fig. 3b). The geothermal systems used for comparison have differing geology, which may influence the temperature distribution at each site. Despite such site-specific features, the general trend is well captured by our model.

### Injection phase

**Fig. 4:**
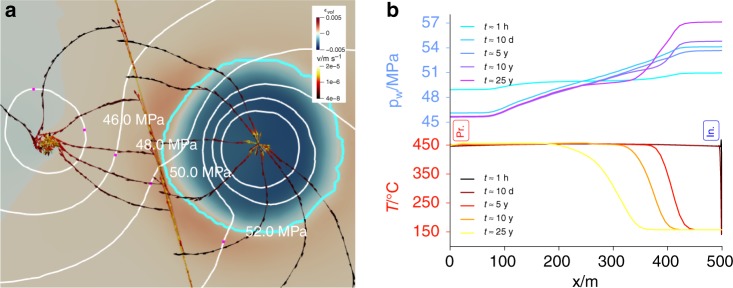
Pressure distribution between the wells and deformation. **a** The streamlines go from the injection well to the extraction well and the volumetric strain follows the thermal field after 25 years of injection. The cyan line indicates the liquid front, which is close to the fault. **b** The pressure distribution is asymmetrical in the long-term and the mid-point moves with time towards the injection well because of the higher viscosity of the liquid water around the injection well due to thermal quenching

Cold water is re-injected during the exploitation phase of geothermal systems, which redirects locally the initial vertically density-driven flow to a horizontal flow from the injection to the production well (Fig. 4a). Stream lines velocity is three orders of magnitude higher than that in the natural convection system. Water accelerates along the more permeable fault, presenting an upward flow because of the heat gradient and the subsequent buoyant forces related to the lower water density in the lower part of the model. The equivalent permeability of the reservoir, accounting for the stimulated region around the wells, is computed with the Cooper–Jacob method and is ${{\bf{k}}}_{\text{eq}}{\,}={\,}1.74{\,}\times{\,} 1{0}^{-15}{\bf{I}}{\,} {{\rm{m}}}^{2}$, slightly higher than initial. Pressure changes are limited to 10 MPa increase and 2 MPa decrease. The gradient is lower within a 100-m radius from the wells because of the higher permeability. The pressure distribution is symmetric between the two wells in the early stages of injection, with the neutral point located in the mid-point, i.e., in the fault. In the long-term, the pressure distribution becomes asymmetrical and the neutral point shifts towards the injection well because the injected cold water has a higher viscosity that increases the head losses around the injection well (see Fig. 4b). Water transitions from SC to liquid phase as the temperature drops (cyan line), but does not evaporate because the pressure remains above 22.064 MPa. After 25 years of exploitation, the liquid water front in the reservoir has reached the fault. While the fluid flow quickly establishes the pressure gradient between the wells, the thermal diffusion is a slower process which generates an additional time dependency in the problem.

### Fault stability and induced seismicity

The injection of cold water cools down and contracts the rock around the well (Fig. 4a), causing negative volumetric strain ${\epsilon }_{\text{vol}}{\,}={\,}{\rm{tr}}{\,} \left({\boldsymbol{\epsilon }}\right){\,}< {{\,}}0$ over an area that increases with time following thermal diffusion. This circular shape is not centred in the injection well because cold water tends to sink as a result of its higher density. Deformation is negligible surrounding the production well, which is in isothermal conditions, hinting towards the dominant effect of thermal changes in the geomechanical response of the reservoir.

**Fig. 5:**
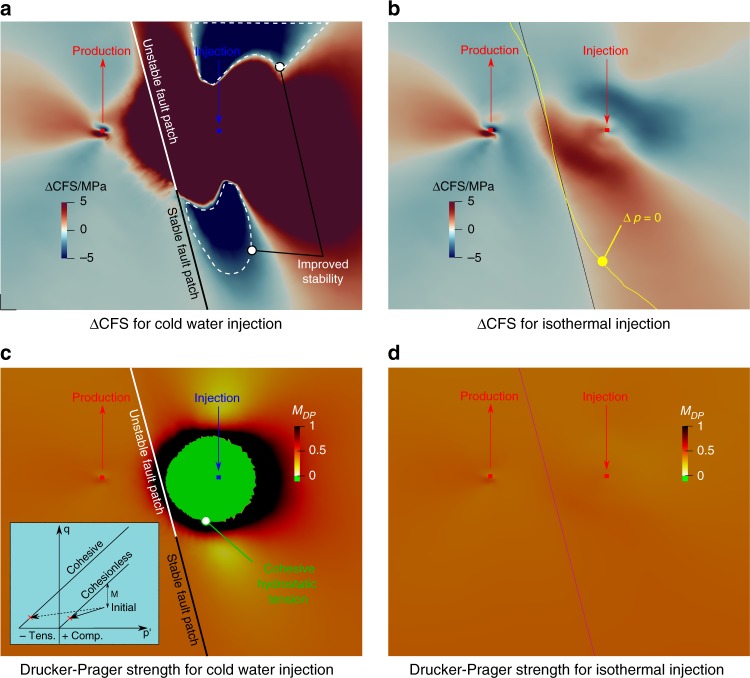
Fault reactivation and reservoir stability. Coulomb Failure Stress changes ($\Delta {\rm{CFS}}$) after 25 years for the cold water (**a**) and isothermal injection (**b**) cases and Drucker-Prager mobilised failure ratio ${M}_{\text{DP}}$ after 25 years for the cold water (**c**) and isothermal injection (**d**) cases. Negative values of ${M}_{\text{DP}}$ imply ${\sigma }_{\text{m}}^{{\prime} }\,\le\, -{c}^{{\prime} }/\tan \,\phi$: hydrostatic tension failure will be reached and tensile fractures will be likely to appear, as highlighted by the schematic representation in the inset in **c**

After 25 years of cold water re-injection, $\Delta {\rm{CFS}}{\,}< {{\,}}0$ (stability increase) occurs above the production well and above and below the cooled region around the injection well as a consequence of a more compressive normal stress and reduced shear stress (Fig. 5a). $\Delta {\rm{CFS}}{\,}> {\,}0$ (stability decrease) occurs between the injection and production well: cooling contracts the rock and induces a normal stress reduction that drives the fault toward failure conditions. The yellow line in Fig. 5b represents the boundary of null pore pressure variation after 25 years of isothermal injection: pore pressure increases are located on the right of the yellow line (injection side), while pore pressure decrease are located to the left of the yellow line (production side). In isothermal injection, flow behaviour dominates instability, which in turn migrates downward as a consequence of the pore pressure increment and normal effective stress reduction. The lower magnitude of $\Delta$CFS in the isothermal injection implies that pore pressure effects play a smaller role than thermal effects in terms of fault stability.

${M}_{\text{DP}}$ values indicate that the rock is failing in two potential ways: when ${M}_{\text{DP}}{\,}> {\,}1.0$, frictional-compressive failure is expected and for ${M}_{\text{DP}}{\,}< {{\,}}0$, the state of stress exceeds hydrostatic tension and tensile failure is most likely (Fig. 5c and d). Tensile failure is a consequence of the rock thermal contraction as it roughly follows the cooled down area. The inset in Fig. 5c provides a schematic representation of the stress paths that lead to tensile failure and compares the here assumed case of cohesive strength with an hypothetical cohesionless one. For isothermal injection, a much smaller portion of the reservoir exhibits increase in the mobilised shear, which remains far from failure ${M}_{\text{DP}}{\,}< {{\,}}1.0$. As expected, no tensile mobilisation is observed because the pressure increase does not drive the rock toward tensile failure, which would essentially mean hydro-fracturing the rock.

**Fig. 6:**
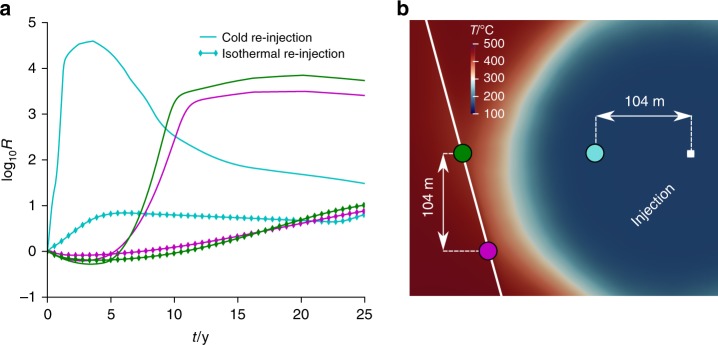
Rate of seismic production. The rate of seismicity production is computed at different locations in the main fault and in the fractured rock (curve colours in **a** refers to points which are indicated in the model in **b**), for isothermal and cold water re-injection. Injecting cold fluids generates significantly higher induced seismicity than injecting fluids in thermal equilibrium with the reservoir

The effective stress changes caused by pressure and temperature variations alter the seismicity rate. Figure. 6 shows the rate of seismicity production in logarithmic scale at the fault and in the fractured rock for cold water injection and isothermal injection. The seismicity rate increases along the fault in both cases. However, the rate of seismic increase within the main fault is significantly enhanced when cooling occurs. Seismicity rate increase is observed in the lower part of the fault after a bit more than 5 years, but is quickly reverted as cooling effects are dominant. The reversion of seismicity from lower to higher parts of the fault is slower when no cooling occurs and only pore pressure changes act in the system. While the seismicity rate in the fault increases almost one order of magnitude for the isothermal injection, there is a considerable four orders of magnitude increase for cold water injection. It should be noted that the seismicity rate increases quickly between 5 and 10 years; as it will be shown in the next paragraph, this corresponds to a time when the mobilised strength in the fault is still not at peak and the cooling front has not reached the fault yet. The observation point in the fractured rock is closer to the injection well, which implies greater thermal stress changes and hence increased induced seismicity in earlier times compared to the further away fault. Overall, cooling has important effects on the rate of seismicity production.

**Fig. 7:**
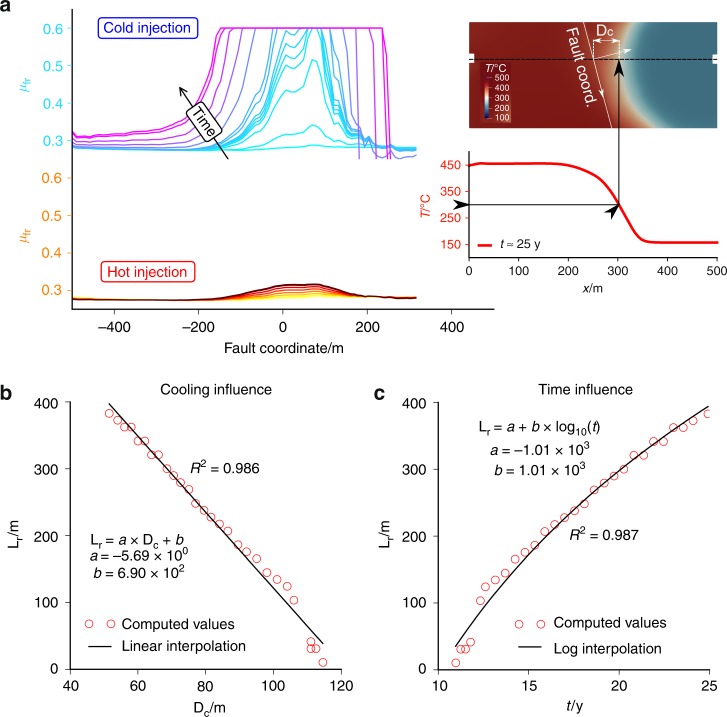
Fault mobilisation. **a** Evolution with time of mobilised friction ${\mu }_{{\rm{fr}}}$ along the fault coordinate for cold water and isothermal injection. **b**, **c** The fault mobilised length ${L}_{{\rm{r}}}$ (${\mu }_{{\rm{fr}}}\,\ge\, 0.6$) of the cold water injection has a linear dependence on the distance of the mean temperature ($T\,=\,30{0}\,^{\circ}{\rm{C}}$) front ${D}_{{\rm{c}}}$ (**b**) and a logarithmic dependence on time $t$ (**c**)

Fault mobilisation is assumed whenever ${\mu }_{{\rm{fr}}}\ge 0.6$^63^ and its evolution with time is shown in Fig. 7a for both scenarios. In isothermal conditions, the mobilised friction in the fault never exceeds critical values and it can be considered stable throughout the whole simulation. On the contrary, for cold water injection, the fault undergoes progressively larger mobilisation size. The size of the unstable patch ${L}_{{\rm{r}}}$ increases with time as the thermal front approaches the fault. Because rock contraction acts as a long-range mechanism to destabilise the fault, the temperature change in the fault alone would not provide sufficient insight. To account for this effect, we computed the point at which the temperature decrease halfway, i.e., the point at which $T{\,}={\,}300$ °C. The point of average temperature migrates with time during thermal diffusion and its distance from the fault along the line connecting the two wells is ${D}_{{\rm{c}}}$ (Fig. 7a). We have plot ${D}_{{\rm{c}}}$ vs. ${L}_{{\rm{r}}}$ and found that a linear relation exists between this two values, as the length of the stress redistribution induced by cooling is proportional to the radius of the cooled region (Fig. 7b). The size of the mobilised fault patch ${L}_{{\rm{r}}}$ is instead proportional to the logarithm of time $\propto {\,}{\mathrm{log}}_{10}{\,}\left(t\right)$, which is a consequence of the thermal diffusion process (Fig. 7c).

## Discussion

SC geothermal systems are located near thermal instabilities (heat plumes) and convective cells establish upward fluid migration and a sub-hydrostatic pressure in the deeper part of the reservoir as a consequence of the reduced fluid density. Estimates of the temperature and pressure distribution are of great help for preliminary assessment of geothermal potential and for project design. Thus, through numerical modelling, concurring hypotheses can be tested with the aim of better locating potential SC resources. Proper assessment of resources and initial conditions is fundamental to design geothermal exploitation systems, from conception to decommissioning. Numerical modelling can be applied to specific geothermal sites, accounting for local geological structures and physical parameters of the rocks.

During cold water re-injection, two concomitant time-dependent processes are active: a fast ($\propto{\,} 1{0}^{6}$ s) diffusion process that counteracts the pore pressure gradient generated by the forced flow, and a slow ($\propto {\,}1{0}^{8}$ s) advection process that transports heat in the fractured rock. The mechanical behaviour of the reservoir is influenced by these two processes, the short-term time dependence is governed by fluid flow and the long-term one by thermal effects. Viscosity increases during cold water re-injection, which in turn increases the total water head and shifts the null-pressure point towards the injection well (Fig. 4). Sinking effects are evident and the liquid water front extends asymmetrically around the injection well. The liquid front reaches the fault after roughly 25 years of injection, implying that SC fluid will still be produced. We have assumed a circular shape of the stimulated area around the wells because stimulation with supercritical water forms a cloud of increased permeability instead of a single planar fracture^27^. Nevertheless, hydrofracturing and hydroshearing with supercritical water is still a poorly understood topic. Different permeability structures around the wells will affect timing and orientation of the thermal front through advective processes, but the general flow conditions will not change in comparison to the current assumptions.

Cooling increases the failure potential and the number of recorded seismic events in both the fractured rock and the main fault. While instabilities in the fractured rock are expected to produce microseismicity, as the existing fractures have limited extent and hence likewise limited rupture area, greater events are associated with reactivation of the main fault that will start slipping after roughly 10 years. Tensile failure is a consequence of thermal contraction and might have a beneficial effect by increasing the permeability, but can also greatly enhance the rate of microseismicity by reducing the mean normal stress acting on the fractures. What is likely to happen is an increase of frequency in small earthquakes in connection with cold water injection during early time. Larger events are delayed and their timing depends essentially on the distance between the cooling front and the existing major fault/s, with important consequences in terms of reservoir management and lifetime assessment. This pattern has been observed at The Geysers, where the number of seismic event closely correlates with injection history^64^.

The magnitude of the seismic events depends on the critical nucleation length in quasi-static regime^65^, which is controlled by frictional weakening associated with slip and dynamic effects of melting and pressurisation^66,67^. For injection-induced seismicity, nucleation size and dynamic fault rupture arrest strongly depend on pore pressure increment in the fault and on the acting background stress state^35^. It is therefore difficult to compute the magnitude of the larger events that involve the slip into the main fault, due to the quasi-static approach adopted here. Moment magnitude estimates can be obtained by enhancing the modelling approach and including discontinuous fault displacement in the finite element formulation with frictional weakening, and eventually coupling the analyses with a dynamic solver to estimate runaway ruptures and propagating crack fronts in dynamic conditions^68,69^. Nevertheless, we have shown how the mobilised size of the fault increases logarithmically with time as the cooling front approaches it. The size of the fault mobilisation is quite large and, as an example, after 25 years of injection has reached $\approx 400$ m in length and, considering the $300$ m of open well section for injection, could have a surface in the order of $S{\,}\approx{\,} 1{0}^{5}{\,} {{\rm{m}}}^{2}$. Thus, large events triggered by cooling of the reservoir are possible and their timing is controlled by the thermal diffusion process.

Our approach is a first attempt to model the complex geomechanical behaviour of ESGS. Several open questions remain at present to be addressed, and future studies should focus on the influence of inelastic behaviour (rock strength decays at high temperature^70^), on the role of geological structures and multiple faults, on the full three dimensional reservoir behaviour, on the presence of fluid mixtures (${{\rm{H}}}_{2}{\rm{O}}{\hbox{--}}{{\rm{CO}}}_{2}{\hbox{--}}{\rm{NaCl}}$), on modelling the frictional behaviour of faults and dynamic rupture. Nevertheless, we have provided an important piece of evidence to support the role of cooling in connection to induced seismicity and fault destabilization. The consequences must be evaluated on individual cases and holistic risk assessment approaches will provide estimates of potential damage, infrastructure disruption and disturbance to the local population. State-of-the-art numerical analyses are a fundamental tool to address the complexities involved in ESGS: entangled with monitoring techniques, they provide precious insights into the physical behaviour of ESGS and will prove essential for long-term safe reservoir management.

## Methods

### Porosity evolution

We report here the detailed derivation of mass, energy and momentum balance equations of porous media leading to the complete description of thermo-hydro-mechanical (THM) coupled processes presented in Eq. (1). Subscripts ${\rm{w}}$ stand for water, ${\rm{s}}$ for solid and ${\rm{m}}$ for porous medium. The sign convention of stresses and strains is that of solid mechanics, i.e., positive is for tensile. The equations are formulated as a function of the primary variables of pore pressure, $p$, temperature, $T$ and displacement vector, ${\bf{u}}$. The mass balance of the solid skeleton writes

3 $$\frac{{{\rm{d}}}_{{\rm{s}}}}{{\rm{d}}t}{\,}\left[\left(1-n\right){\,}{\rho }_{\text{s}}\right]{\,}+{\,}(1{\,}-{\,}n){\rho }_{\text{s}}\nabla{\,} \cdot{\,} {{\bf{v}}}_{\text{s}}{\,}={\,}0,$$

where $n$ is porosity, ${\rho }_{\text{s}}$ is solid density, ${{\bf{v}}}_{\text{s}}$ is the solid’s velocity, $t$ is time and the operator ${{\rm{d}}}_{{\rm{s}}}/{\rm{d}}t$ represents the material time derivative of the solid defined as

4 $$\frac{{{\rm{d}}}_{\text{s}}{\rho }_{\text{s}}}{{\rm{d}}t}{\,}={\,}\frac{\partial {\rho }_{\text{s}}}{\partial t}{\,}+{\,}{{\bf{v}}}_{\text{s}}{\,}\cdot{\,} \nabla {\rho }_{\text{s}}.$$

Expanding Eq. (3) yields an expression for the rate of change in porosity

5 $$\frac{{{\rm{d}}}_{{\rm{s}}}n}{{\rm{d}}t}{\,}={\,}(1{\,}-{\,}n){\,}\frac{1}{{\rho }_{\text{s}}}\frac{{{\rm{d}}}_{{\rm{s}}}{\rho }_{\text{s}}}{{\rm{d}}t}{\,}+{\,}(1{\,}-{\,}n)\nabla{\,} \cdot{\,} {{\bf{v}}}_{\text{s}}{\,}={\,}(1{\,}-{\,}n){\,}\left(\frac{1}{{\rho }_{\text{s}}}\frac{{{\rm{d}}}_{{\rm{s}}}{\rho }_{\text{s}}}{{\rm{d}}t}{\,}+{\,}{\dot{\epsilon }}_{\text{v}}\right),$$

where ${\dot{\epsilon }}_{\text{v}}$ is the volumetric strain rate. The solid density can be expressed as a function of temperature $T$, mean effective stress $\sigma ^{\prime}_{\rm{m}} {\,}={\,}{\rm{tr}}{\,} \left({\boldsymbol{\sigma }}^{\prime} \right){\,}/3$ and pore pressure $p$^71^ such that applying chain derivation yields

6 $$\frac{1}{{\rho }_{\text{s}}}\frac{{{\rm{d}}}_{{\rm{s}}}{\rho }_{\text{s}}}{{\rm{d}}t}{\,}={\,}\frac{1}{{\rho }_{\text{s}}}\frac{\partial {\rho }_{\text{s}}}{\partial T}\frac{{\rm{d}}_{\rm{s}}T}{{\rm{d}}t}{\,}+{\,}\frac{1}{{\rho }_{\text{s}}}\frac{\partial {\rho }_{\text{s}}}{\partial \sigma ^{\prime}_{\rm{m}} }\frac{{\rm{d}}_{\rm{s}}\sigma ^{\prime}_{\rm{m}} }{{\rm{d}}t}{\,}+{\,}\frac{1}{{\rho }_{\text{s}}}\frac{\partial {\rho }_{\text{s}}}{\partial p}\frac{{\rm{d}}_{\rm{s}}p}{{\rm{d}}t},$$

with linear thermal expansion coefficient

7 $$3{\alpha }_{\text{s}}{\,}={\,}-\frac{1}{{\rho }_{\text{s}}}\frac{\partial {\rho }_{\text{s}}}{\partial T},$$

and compressibility of the solid skeleton to pore pressure changes

8 $$\frac{1}{{\rho }_{\text{s}}}\frac{\partial {\rho }_{\text{s}}}{\partial p}{\,}={\,}\frac{1}{{K}_{\text{s}}},$$

where ${K}_{\text{s}}$ is the intrinsic bulk modulus of the solid phase. The compressibility of the solid phase in response to effective stress changes writes

9 $$\frac{1}{{\rho }_{\text{s}}}\frac{\partial {\rho }_{\text{s}}}{\partial \sigma ^{\prime}_{\rm{m}} }{\,}={\,}-\frac{1}{\left(1{\,}-{\,}n\right){\,}{K}_{\text{s}}},$$

so that the solid density constitutive equation becomes

10 $$\frac{1}{{\rho }_{\text{s}}}\frac{{{\rm{d}}}_{{\rm{s}}}{\rho }_{\text{s}}}{{\rm{d}}t}{\,}={\,}\frac{1}{{K}_{\text{s}}}\frac{{\rm{d}}_{\text{s}}p}{{\rm{d}}t}{\,}-{\,}3{\alpha }_{\text{s}}\frac{{\rm{d}}_{\text{s}}T}{{\rm{d}}t}{\,}-{\,}\frac{1}{\left(1{\,}-{\,}n\right){\,}{K}_{\text{s}}}\frac{{\rm{d}}_{\text{s}}\sigma ^{\prime}_{\rm{m}} }{{\rm{d}}t}.$$

The effective stress acting between the grains due to volumetric strains is reduced by grain volume changes due to thermal effects and due to compression in response to fluid pressure

11 $$\frac{{\rm{d}}_{\text{s}}\sigma ^{\prime}_{\rm{m}} }{{\rm{d}}t}{\,}={\,}K{\,}\left(\frac{1}{{K}_{\text{s}}}\frac{{\rm{d}}_{\text{s}}p}{{\rm{d}}t}{\,}-{\,}3{\alpha }_{\text{s}}\frac{{\rm{d}}_{\text{s}}T}{{\rm{d}}t}{\,}+{\,}\frac{{\rm{d}}_{\text{s}}{\epsilon }_{\text{v}}}{{\rm{d}}t}\right),$$

where *K* is the drained bulk modulus and Biot’s coefficient is defined as

12 $$\alpha {\,}={\,}1-\frac{K}{{K}_{\text{s}}}.$$

Eq. (6) can now be recombined into the constitutive equation of the solid grain density, yielding^71^

13 $$\frac{1}{{\rho }_{\text{s}}}\frac{{\rm{d}}_{{\rm{s}}}{\rho }_{\text{s}}}{{\rm{d}}t}{\,}={\,}\frac{1}{1{\,}-{\,}n}\left[\frac{\alpha {\,}-{\,}n}{{K}_{\text{s}}}\frac{{\rm{d}}_{\text{s}}p}{{\rm{d}}t}{\,}-{\,}3{\alpha }_{\text{s}}{\,}\left(\alpha {\,}-{\,}n\right)\frac{{\rm{d}}_{\text{s}}T}{{\rm{d}}t}{\,}-{\,}\left(1{\,}-{\,}\alpha \right){\,}\frac{{\rm{d}}_{\text{s}}{\epsilon }_{\text{v}}}{{\rm{d}}t}\right].$$

Introducing Eq. (13) into Eq. (5), the final expression of porosity variation becomes

14 $$\frac{{{\rm{d}}}_{{\rm{s}}}n}{{\rm{d}}t}{\,}={\,}\left(\alpha{\,} -{\,}n\right){\,}\left(\frac{1}{{K}_{\text{s}}}\frac{{\rm{d}}_{\text{s}}p}{{\rm{d}}t}{\,}-{\,}3{\alpha }_{\text{s}}\frac{{\rm{d}}_{\text{s}}T}{{\rm{d}}t}{\,}+{\,}\frac{{\rm{d}}_{\text{s}}{\epsilon }_{\text{v}}}{{\rm{d}}t}\right).$$

### Mass balance equation

Recalling Eq. (5), the mass balance of the solid skeleton writes

15 $$\left(1{\,}-{\,}n\right)\nabla {\,}\cdot{\,} {{\bf{v}}}_{\text{s}}{\,}+{\,}\left(1{\,}-{\,}n\right){\,}\frac{1}{{\rho }_{\text{s}}}\frac{{{\rm{d}}}_{{\rm{s}}}{\rho }_{\text{s}}}{{\rm{d}}t}{\,}-{\,}\frac{{{\rm{d}}}_{{\rm{s}}}n}{{\rm{d}}t}{\,}={\,}0.$$

The mass balance of the fluid flow writes

16 $$n\nabla{\,} \cdot{\,} {{\bf{v}}}_{\text{w}}{\,}+{\,}n\frac{1}{{\rho }_{\text{w}}}\frac{{{\rm{d}}}_{{\rm{w}}}{\rho }_{\text{w}}}{{\rm{d}}t}{\,}+{\,}\frac{{{\rm{d}}}_{{\rm{w}}}n}{{\rm{d}}t}{\,}={\,}{Q}_{{\rm{H}}}.$$

where ${{\bf{v}}}_{\text{w}}$ is the fluid velocity and ${Q}_{{\rm{H}}}$ is the source term. Recalling the definition of material derivative in Eq. (4), we can write the transformation between material derivatives with respect to the fluid phase ${{\rm{d}}}_{{\rm{w}}}/{\rm{d}}t$ to material derivatives with respect to the solid phase ${{\rm{d}}}_{{\rm{s}}}/{\rm{d}}t$, i.e., for a given quantity $a$

17 $$\frac{{{\rm{d}}}_{{\rm{w}}}a}{{\rm{d}}t}{\,}={\,}\frac{{{\rm{d}}}_{{\rm{s}}}a}{{\rm{d}}t}{\,}+{\,}\left({{\bf{v}}}_{\text{w}}{\,}-{\,}{{\bf{v}}}_{\text{s}}\right){\,}\cdot{\,} \nabla a.$$

The fluid balance equation can then be rewritten as

18 $$n\nabla{\,} \cdot{\,} {{\bf{v}}}_{\text{w}}{\,}+{\,}n\frac{1}{{\rho }_{\text{w}}}\frac{{{\rm{d}}}_{{\rm{w}}}{\rho }_{\text{w}}}{{\rm{d}}t}{\,}+{\,}\frac{{{\rm{d}}}_{{\rm{s}}}n}{{\rm{d}}t}+\left({{\bf{v}}}_{\text{w}}{\,}-{\,}{{\bf{v}}}_{\text{s}}\right){\,}\cdot{\,} \nabla n{\,}={\,}{Q}_{{\rm{H}}}.$$

Substituting the expression of porosity material derivative from Eq. (18) into solid mass balance Eq. (15), with the hypothesis of small gradients and re-arranging, yields

19 $$n\frac{1}{{\rho }_{\text{w}}}\frac{{{\rm{d}}}_{{\rm{w}}}{\,}{\rho }_{\text{w}}}{{\rm{d}}t}{\,}+{\,}\left(1{\,}-{\,}n\right)\frac{1}{{\rho }_{\text{s}}}\frac{{{\rm{d}}}_{{\rm{s}}}{\,}{\rho }_{\text{s}}}{{\rm{d}}t}{\,}+{\,}n\nabla{\,} \cdot{\,} {{\bf{v}}}_{\text{w}}{\,}+{\,}\left(1{\,}-{\,}n\right)\nabla{\,} \cdot{\,} {{\bf{v}}}_{\text{s}}{\,}={\,}{Q}_{{\rm{H}}}.$$

The density of water depends on pressure $p$ and temperature $T$, so that its constitutive equation writes

20 $$\frac{1}{{\rho }_{\text{w}}}\frac{{{\rm{d}}}_{{\rm{w}}}{\,}{\rho }_{\text{w}}}{{\rm{d}}t}{\,}={\,}\frac{1}{{\rho }_{\text{w}}}\frac{\partial {\rho }_{\text{w}}}{\partial p}\frac{{\rm{d}}_{\rm{w}}p}{{\rm{d}}t}{\,}+{\,}\frac{1}{{\rho }_{\text{w}}}\frac{\partial {\rho }_{\text{w}}}{\partial T}\frac{{\rm{d}}_{\rm{w}}T}{{\rm{d}}t}{\,}={\,}{\beta }_{\text{w}}\frac{{\rm{d}}{\rm{w}}p}{{\rm{d}}t}{\,}-{\,}{\alpha }_{\text{w}}\frac{{\rm{d}}_{\rm{w}}T}{{\rm{d}}t},$$

where ${\beta }_{\text{w}}$ is water compressibility and ${\alpha }_{\text{w}}$ is water volumetric thermal expansion coefficient. The seepage velocity is ${\bf{v}}{\,}={\,}n{\,}\left({{\bf{v}}}_{\text{w}}{\,}-{\,}{{\bf{v}}}_{\text{s}}\right)$ and relates to pressure changes through Darcy’s law

21 $${\bf{v}}{\,}= \, -\frac{{\bf{k}}}{{\mu }_{{\rm{w}}}}{\,}\left(\nabla p{\,}-{\,}{\rho }_{{\rm{w}}}{\bf{g}}\right),$$

where ${\bf{k}}$ is the intrinsic permeability tensor, ${\mu }_{{\rm{w}}}$ is the water dynamic viscosity and ${\bf{g}}$ is the body force vector (only gravity is present in this case). Recalling that volumetric strain rate is ${\dot{\epsilon }}_{\text{v}}{\,}={\,}\nabla {\,}\cdot{\,} {{\bf{v}}}_{\text{s}}$ and the expression of solid compressibility Eq. (13), the continuity equation of the porous medium Eq. (19) can be rearranged into

22 $$\left(n{\beta }_{\text{w}}{\,}+{\,}\frac{\alpha {\,}-{\,}n}{{K}_{\text{s}}}\right){\,}\frac{{\rm{d}}_{\rm{s}}p}{{\rm{d}}t}{\,}-{\,}\left[n{\alpha }_{\text{w}}{\,}+{\,}3{\,}\left(n{\,}-{\,}1\right){\,}{\alpha }_{\text{s}}\right]{\,}\frac{{\rm{d}}_{\rm{s}}T}{{\rm{d}}t}{\,}+{\,}\nabla {\,}\cdot{\,} {\bf{v}}+\alpha {\dot{\epsilon }}_{\text{v}}{\,}={\,}{Q}_{{\rm{H}}}.$$

The first coefficient of Eq. (22) represents the storage term $S$, while the second coefficient describes the average thermal expansion of the porous medium. Together with the volumetric strain term, fluid-solid differential thermal expansion effects are captured.

### Energy balance equation

Advective and conductive heat transfer in the porous medium are derived from the heat balance equation

23 $${(c\rho)}_{{\rm{m}}}{\,}\frac{{\rm{d}}_{\rm{s}}T}{{\rm{d}}t}{\,}-{\,}\nabla {\,}\cdot{\,} \left({{\boldsymbol{\lambda }}}_{{\rm{m}}}\nabla T\right){\,}+{\,}{\rho }_{{\rm{w}}}{c}_{{\rm{w}}}{\bf{v}}{\,}\cdot{\,} \nabla T{\,}={\,}{Q}_{{\rm{T}}},$$

where $c$ is specific heat and ${\boldsymbol{\lambda }}$ is the thermal conductivity, which for porous media write

24 $${(c\rho)}_{{\rm{m}}}{\,}={\,}n\ {\rho }_{{\rm{w}}}\ {c}_{{\rm{w}}}{\,}+{\,}(1{\,}-{\,}n){\,}{\rho }_{{\rm{s}}}\ {c}_{{\rm{s}}},$$

and

25 $${{\boldsymbol{\lambda }}}_{{\rm{m}}}{\,}={\,}n{\lambda }_{{\rm{w}}}{\bf{I}}{\,}+{\,}(1-n){\,}{{\boldsymbol{\lambda }}}_{{\rm{s}}},$$

where ${\bf{I}}$ is the identity matrix. The first term of Eq. (23) represents the heat storage term, the second term is related to conductive heat transport via Fourier’s law and the third term is the advective heat transport term. The right-hand side ${Q}_{{\rm{T}}}$ is a thermal source term. Thermal effects due to deformation have been neglected.

### Linear momentum balance equation

The linear momentum balance of the porous medium writes

26 $$\nabla {\,}\cdot{\,} {\boldsymbol{\sigma }}{\,}+{\,}{\rho }_{{\rm{m}}}{\bf{g}}{\,}={\,}{\bf{0}}.$$

The first term is the divergence of Cauchy’s second order total stress tensor ${\boldsymbol{\sigma }}$ and the second term represents body forces due to gravity. The effective medium density is expressed as

27 $${\rho }_{{\rm{m}}}{\,}={\,}n{\rho }_{{\rm{w}}}{\,}+{\,}(1{\,}-{\,}n){\,}{\rho }_{{\rm{s}}},$$

and the stress acting exclusively on the solid skeleton is Biot’s effective stress ${\boldsymbol{\sigma }}^{\prime}$ which writes

28 $${\boldsymbol{\sigma }}^{\prime} {\,}={\,}{\boldsymbol{\sigma }}{\,}+{\,}\alpha p{\bf{I}}.$$

The constitutive equation of the thermo-elastic solid writes

29 $${\boldsymbol{\sigma }}^{\prime} {\,}={\,}K\left({\rm{tr}}\ {\boldsymbol{\epsilon }}{\,}-{\,}3{\alpha }_{{\rm{s}}}\Delta T\right){\,}{\bf{I}}{\,}+{\,}2G{{\boldsymbol{\epsilon }}}^{{\rm{D}}},$$

where *K* and $G$ are the bulk and shear moduli of elasticity, respectively, the infinitesimal strain tensor ${\boldsymbol{\epsilon }}$ is the symmetric part of the displacement gradient

30 $${\boldsymbol{\epsilon }}{\,}={\,}\frac{1}{2}\left[\nabla {\bf{u}}{\,}+{\,}{\left(\nabla {\bf{u}}\right)}^{{\rm{T}}}\right],$$

and ${{\boldsymbol{\epsilon }}}^{{\rm{D}}}$ is the deviator of the strain tensor. Other elastic constants can be employed, such as Young’s modulus, $E$, and Poisson’s ratio, $\nu$.

### Fluid compressibility and thermal expansion

We have applied a finite difference scheme to compute the water's compressibility

31 $${\beta }_{\text{w}}{\,}\left(p,T\right){\,}={\,}\frac{1}{{\rho }_{\text{w}}}{\frac{\partial {\rho }_{\text{w}}}{\partial p} \bigg |}_{T}{\,}={\,}\frac{1}{{\rho }_{\text{w}}{\,}\left(p,T\right)}\frac{{\rho }_{\text{w}}{\,}\left(p{\,}+{\,}\delta p,T\right){\,}-{\,}{\rho }_{\text{w}}{\,}\left(p,T\right)}{\delta p},$$

and thermal expansion

32 $${\alpha }_{\text{w}}{\,}\left(p,T\right){\,}={\,}-{\,}\frac{1}{{\rho }_{\text{w}}}{\frac{\partial {\rho }_{\text{w}}}{\partial T} \bigg|}_{p}{\,}={\,}-{\,}\frac{1}{{\rho }_{\text{w}}{\,}\left(p,T\right)}\frac{{\rho }_{\text{w}}{\,}\left(p,T{\,}+{\,}\delta T\right){\,}-{\,}{\rho }_{\text{w}}{\,}\left(p,T\right)}{\delta T}.$$

### Permeability distribution

The fracture density around the two wells follows a spline bell-shape distribution

33 $$\omega {\,}={\,} \left\{\begin{array}{lcc}\left(1{\,}-{\,}{\omega }_{1}\right){\,}{\left(1{\,}-{\,}\frac{{r}^{2}}{{R}_{{\rm{p}}}^{2}}\right)}^{2}{\,}+{\,}{\omega }_{1}&{\,}r{\,}\le{\,} {R}_{{\rm{p}}}\\ {\omega }_{1}&{\,}r{\,}> {\,}{R}_{{\rm{p}}}\end{array}\right.$$

where ${R}_{{\rm{p}}}=100$ m and ${\omega }_{1}$ is taken as a Weibull distribution

34 $${\omega }_{1}{\,}={\,}\left\{\begin{array}{lcc}\frac{\hat{k}}{{\omega }_{0}}{\,}{(\frac{x}{\hat{k}})}^{\hat{k}{\,}-{\,}1}\exp {\,}(-{(\frac{x}{{\omega }_{0}})}^{\hat{k}}){\,}&{\,}x{\,}\ge{\,} 0\\ 0{\,}&{\,}x{\,}< {{\,}}0\end{array}\right.$$

with $${\omega }_{0}{\,}={\,}0.25$$ and $$\hat{k}{\,}={\,}10$$.

### Numerical implementation

The spatial discretization is achieved with the Galerkin method and the temporal discretization is carried out with an implicit finite difference scheme^72,73^. The weak forms of the balance equations write, for the mass

35 $$\begin{array}{lcc} \int _{\Omega }{\,}S\frac{\partial p}{\partial t}\psi d\Omega {\,}+{\,} \int _{\Omega }{\,}{k}_{{\rm{ws}}}\frac{\partial T}{\partial t}\psi d\Omega {\,}+{\,} \int _{\Omega }{\,}\nabla {\psi }^{\text{T}}{\,}\cdot{\,} {\bf{v}}{d}\Omega \\ {\,}+{\,} \int _{\Omega }\alpha \psi{\,} \left(\nabla {\,}\cdot{\,} \frac{\partial {\bf{u}}}{\partial {t}}\right){\,}d\Omega {\,}+{\,} \int_{\Gamma }\psi {{\bf{q}}}_{H}{\,}\cdot{\,} {\bf{n}}d\Gamma {\,}-{\,} \int_{\Omega }\psi {Q}_{H}d\Omega {\,}={\,}0,\end{array}$$

heat

36 $$\int_{\Omega }{\,}\psi {c}_{{\rm{m}}}{\rho }_{{\rm{m}}}\frac{\partial T}{\partial t}d\Omega {\,}-{\,} \int_{\Omega }{\,}\nabla {\psi }^{\text{T}}{\,}\cdot{\,} {{\bf{q}}}_{{\rm{T}}}d\Omega {\,}+{\,} \int_{\Gamma }{\,}\psi {{\bf{q}}}_{{\rm{T}}}{\,}\cdot{\,} {\bf{n}}d\Gamma {\,}-{\,} \int_{\Omega }{\,}\psi {Q}_{{\rm{T}}}d\Omega {\,}={\,}0,$$

and momentum

37 $$\int_{\Omega }{\,}\frac{1}{2}{\,}\left({\boldsymbol{\sigma }}^{\prime} {\,}-{\,}\alpha p{\bf{I}}\right){\,}:{\,}\left[\nabla \psi {\,}+{\,}{\left(\nabla \psi \right)}^{\text{T}}\right]{\,}d\Omega {\,}\\ - \int_{\Omega }{\psi }^{\text{T}}{\,}\cdot{\,} \rho {\bf{g}}d\Omega {\,}-{\,} \int_{\Gamma }{\psi }^{\text{T}}\cdot \left({\boldsymbol{\sigma }}{\,}\cdot{\,} {\bf{n}}\right){\,}d\Gamma {\,}={\,}0,$$

where ${q}_{{\rm{T}}}{\,}={\,}-{\,}{{\boldsymbol{\lambda }}}_{{\rm{m}}}\nabla T{\,}+{\,}{\rho }_{{\rm{w}}}{c}_{{\rm{w}}}{\bf{v}}T$ groups together the advective and diffusive heat fluxes of the heat equation^73^. Linear test functions $\psi{\,}\in{\,} {V}^{1}{\,}\subset{\,} {H}_{\Gamma }^{1}{\,}{\left(\Omega \right)}^{1}$ are employed for the heat and mass transport equations (TH) and ${\boldsymbol{\psi }}{\,}\in{\,} {V}^{n}{\,}\subset{\,} {H}_{\Gamma }^{1}{\,}{\left(\Omega \right)}^{n}$ is a quadratic test function in $n$-dimensional space applied to the weak form of the momentum balance equation, $\Omega$ denotes the whole problem domain and $\Gamma$ its boundary. The primary variables are approximated by admissible interpolation functions belonging to the Taylor-Hood space of finite elements. The system is re-written as

38 $$\begin{array}{ccc}{{\bf{M}}}_{{\rm{H}}}\dot{{\bf{p}}}{\,}+{\,}{{\bf{K}}}_{{\rm{H}}}{\bf{p}}&{\,}={\,}&{{\bf{f}}}_{{\rm{H}}}\left({\bf{T,u}}\right)\\ {{\bf{M}}}_{{\rm{T}}}\dot{{\bf{T}}}{\,}+{\,}{{\bf{K}}}_{{\rm{T}}}{\bf{T}}&{\,}={\,}&{{\bf{f}}}_{{\rm{T}}}\left({\bf{p}}\right)\\ {{\bf{K}}}_{{\rm{M}}}{\bf{u}}&{\,}={\,}&{{\bf{f}}}_{{\rm{M}}}\left({\bf{T,p}}\right),\end{array}$$

where ${\bf{M}}$ and ${\bf{K}}$ are mass and Laplace matrices, respectively, functions ${\bf{f}}$ denote the right-hand side and the indices indicate the process to which they are specific. The mechanical stiffness matrix writes ${{\bf{K}}}_{{\rm{M}}}{\,}={\,}{\int }_{\Omega }{\,}{{\bf{B}}}^{{\rm{T}}}{\bf{D}}{\bf{B}}{\rm{d}}\Omega$, with the ${\bf{B}}$ containing the gradient of the shape functions ${\bf{N}}$ to calculate the strains ${\boldsymbol{\epsilon }}$. The matrix ${\bf{D}}$ is the constitutive tangent matrix and the shape functions approximate the sought solution ${\bf{u}}$ in terms of its nodal values $\hat{{\bf{u}}}$ as

39 $${\bf{u}}{\,}={\,}{\bf{N}}\hat{{\bf{u}}}.$$

The general form of the mass and Laplace matrices writes

40 $${\bf{M}}{\,}={\,}{\int }_{\Omega }{\,}{\bf{N}}\hat{M}{{\bf{N}}}^{{\rm{T}}}d\Omega,$$

and

41 $${\bf{K}}{\,}={\,}{\int }_{\Omega }{\,}\nabla {\bf{N}}\hat{{\bf{K}}}\nabla {{\bf{N}}}^{{\rm{T}}}d\Omega,$$

where $\hat{M}$ is a scalar material parameter, $\hat{{\bf{K}}}$ is a material tensor and linear or higher order shape function are used based on the process. The solution is achieved through a staggered scheme that combines Newton–Raphson and Picard solution methods^72^.

## Data Availability

Data generated in this study can be requested to the corresponding author. The numerical code employed for the analyses is open source and can be freely downloaded at 10.5281/zenodo.3294169. All data supporting the findings of this study are available within the paper and the methods section. Further information on compiling the code, as well as tutorials and simulation examples about OpenGeoSys are freely accessible at https://www.opengeosys.org/.

## Code availability

The OpenGeoSys full source code employed for the analyses of this paper is available for download at 10.5281/zenodo.3294169. Further information about the code can be found at https://www.opengeosys.org/.
